# Effects of different orchard tree pruning residues on the yield and nutrient composition of *Lentinus edodes*

**DOI:** 10.3389/fnut.2024.1477586

**Published:** 2024-11-29

**Authors:** Yuanyuan Zhang, Quanshun Li, Longfei Duan, Jian Ding, Yue Li, Yong Wang, Hongyan Xu, Baofu Qin

**Affiliations:** ^1^College of Life Sciences, Northwest Agriculture and Forest University, Xianyang, Shaanxi, China; ^2^Institute of Edible Fungi Research, Ankang Academy of Agricultural Sciences, Ankang, Shaanxi, China; ^3^Changqing Forestry Bureau of Shaanxi Province, Hanzhong, Shaanxi, China; ^4^Academy of Agriculture and Forestry Sciences, Qinghai University, Xining, Qinghai, China

**Keywords:** *Lentinus edodes*, orchard tree pruning residues, yield, nutritional quality, protein nutrition evaluation

## Abstract

**Introduction:**

As the scale of *Lentinus edodes* cultivation expands, challenges such as substrate shortages and rising production costs in mushroom cultivation have become increasingly prominent. Fruit tree pruning residue has the potential to serve as an alternative substrate, offering a sustainable solution. This study evaluates the feasibility of incorporating various types of fruit tree pruning residues into *L. edodes* cultivation.

**Methods:**

Different ratios of *Quercus* sawdust (QS), *Malus pumila* pruning (MPP), *Vitis vinifera* pruning (VVP), *Actinidia deliciosa* pruning (ADP), *Ziziphus jujuba* pruning (ZJP), and *Morus alba* pruning (MAP) were tested as substrates. The effects on yield, amino acid profiles, and protein content of *L. edodes* fruiting bodies were analyzed. The control substrate comprised 80% QS, and the experimental groups incorporated varying ratios of fruit tree residues.

**Results:**

Compared with the control, yields increased by 14.86% (QS-MPP), 8.1% (QS-VVP), 18.92% (QS-ZJP), and 22.97% (QS-MAP). The MAP group had 21.21% higher ash content, while the QS-MAP group exhibited the highest crude protein content (10.84% increase). The QS-MPP group showed the highest crude fiber content (1.72 g/100 g). Crude polysaccharide and fat contents in the ZJP group increased by 110.77% and 10.15%, respectively. Mineral content varied, with QS-MPP showing the highest calcium, potassium, manganese, and magnesium levels, and VVP exhibiting the highest iron and copper levels. Amino acid analysis revealed QS-MPP had the highest levels of threonine, valine, isoleucine, serine, cysteine, glycine, and histidine, while QS-VVP had the highest leucine, aspartate, glutamate, and arginine levels. The best formulation was determined as 40% QS, 40% MPP, 17% bran, 1% sucrose, 1% CaCO_3_, and 1% gypsum.

**Discussion:**

These results highlight the potential of fruit tree pruning residues as a sustainable substrate for *L. edodes* cultivation, ensuring high yields and enhanced nutritional quality. This approach can contribute to cost-effective and environmentally friendly mushroomproduction.

## 1 Introduction

Shiitake mushrooms (*Lentinus edodes*) represent the largest global source of edible mushrooms, with dried products being the most common consumption form over a wide range of global markets (1, 2). In 2022, China produced ~12.96 million tons of *L. edodes*, accounting for 98.3% of the total global production of shiitake mushrooms. China is the world's oldest traditional region for shiitake mushroom cultivation (3, 4). Traditionally, sawdust was the primary substrate for cultivating *L. edodes*, typically comprising ~50% synthetic logs or bag substrates (5). *L. edodes* can be cultivated on a variety of lignocellulosic substrates, including coffee pulp, sugarcane bagasse, cereal straw, vineyard pruning, and sorghum stubble (6–10). However, with the rapid expansion of *L. edodes* cultivation, substrate shortages and rising production costs are becoming significant challenges to the industry. Consequently, identifying alternative substrates for *L. edodes* production is an increasingly crucial task.

Annually, ~200 billion tons of organic matter are produced via photosynthesis, with a significant portion classified as agro-industrial waste (11). This situation poses an environmental threat, as such material is often burned or left to decompose naturally (12). Utilizing forest and fruit waste to cultivate edible fungi is a highly effective solution with dual advantages of addressing the raw material shortage in fungi cultivation while enhancing the use of agricultural and forestry waste (13–17). Moreover, this approach maximizes resource utilization, minimizes environmental pollution, and offers significant economic and ecological benefits, promising broad application prospects (10, 18). At the same time, this substrate promotes the quick formation of fruiting bodies and mycelial growth, while also mitigating environmental pollution from pomace (4). In the Shaanxi province of China, sawdust represents only 6.4% of fruit tree branches sold on the market, with up to 40.2% of this potential substrate being accumulated and abandoned, resulting in a serious waste of resources (17). Thus, it is essential to implement the fundamental materialization of fruit tree branches by promoting the use of sawdust to cultivate edible fungi as an eco-friendly method for reusing agricultural waste (19, 20).

The pruning residue from fruit trees is rich in cellulose, lignin, and mineral elements. The nutrient content of such pruning residues is higher than that of corn stalks and many herbaceous plants, making them suitable substrates for cultivating edible fungi (18–20), including the residues from the pruned trees of *Stropharia rugosoannulata, Pleurotus ostreatus, Pleurotus eryngii*, and *Ganoderma lucidum* (21–24). Using fruit tree pruning residue as the main ingredient for cultivating *Pleurotus ostreatus* and *L*. *edodes* was shown to provide substantial amounts of lignin, cellulose, and hemicellulose, which are the primary carbon sources required in the cultivation substrate for edible fungi (17). Sawdust from the cultivation material of *Vitis vinifera* branches can be used as a substrate for cultivating *P. ostreatus*, and the use of appropriate proportions of sawdust from *V. vinifera* branches can shorten the spawning period and increase the contents of zinc, calcium, and selenium in the mushrooms (4). Cultivating *Pleurotus eryngii* and *Hypsizygus marmoreu*s on *V. vinifera* pruning residues led to superior results over traditional cultivation methods, particularly in terms of the total and flavor amino acid contents in the mushrooms (25). However, there is scant research of the impact of the residues of *Actinidia deliciosa, Malus pumila, Morus alba*, and *Ziziphus jujuba* orchards as substrates for the cultivation of *L*. *edodes*, along with limited comprehensive evaluations such as protein assessment.

With the continuous expansion of fruit orchard planting areas in China, the quantity of discarded fruit from tree pruning is increasing. Fruit tree pruning residues contain abundant minerals and are valuable biological resources. However, current improper utilization methods have led to substantial waste of these resources (26). According to data released by the National Bureau of Statistics of China, the area of fruit orchards in China reached 1.265 million hectares in 2020. Therefore, further research is needed to determine optimal strategies for the practical use of fruit tree branches as a valuable resource in agricultural production rather than waste as an important initiative for the development of a green circular agriculture industry. Toward this end, the objective of this study was to assess the feasibility of cultivating *L*. *edodes* on six types of fruit tree pruning residues as raw materials by measuring indicators of mycelial growth and fruiting body development. Additionally, a comparative analysis of the nutritional composition and protein content of *L*. *edodes* grown on the six substrates was conducted to provide a scientific reference for functional mushroom research and to further explore and promote the resource utilization technology of fruit tree pruning residue.

## 2 Materials and methods

### 2.1 Mushroom strain and culture

The *L. edodes* strain Anxiang-2 was provided by the Ankang Academy of Agricultural Sciences and stored at the China General Microbiological Culture Collection Center of Ankang, China (CGMCC 23883). The strain was cultured and preserved in potato dextrose agar (PDA) medium. The PDA was prepared by boiling 200 g peeled potatoes in 1 L of distilled water until soft. The mixture was then strained to obtain the potato extract; 200 mL of the potato extract was combined with 20 g dextrose and 15 g agar. The obtained medium was autoclaved at 121°C for 15–20 min prior to use.

A sterile technique was used to obtain a pure culture by transferring a small piece of the *L. edodes* mycelium from a source culture plate onto the surface of a PDA plate using a sterile loop or needle. The inoculated plates were incubated at 25°C in the dark for 7–10 days, allowing the mycelium to grow and colonize the medium. After incubation, well-isolated colonies exhibiting typical morphological characteristics of *L. edodes* were selected. A small piece of the selected mycelium was then transferred to a new PDA plate to obtain a pure culture as described above; the incubation and selection process was repeated as necessary to ensure purity.

The pure cultures were stored at 4°C for short-term use. For long-term preservation, the strain was sub-cultured on PDA and stored at −80°C in a cryoprotectant (15% glycerol) or preserved in a lyophilized form.

#### 2.1.1 Substrate materials and processing

The study took place at Shaanxi Sanqin Forest and Fungi Industry Ltd. from March 2022 to May 2023. The pruning residues of the following orchard fruit trees were evaluated and compared as potential substrates for *L*. *edodes* cultivation: *Quercus* sp., *Malus pumila, Vitis vinifera, Actinidia deliciosa, Ziziphus jujuba*, and *Morus alba*.

The experiment comprised a total of 11 treatments (Table 1). The raw materials were thoroughly mixed according to the formula, with the moisture content controlled at 60%. After uniform mixing, each 1,200 g dry weight mixture was placed in a polyethylene bag (17 cm × 58 cm × 0.006 cm) and sterilized at 121°C for 4 h. After cooling, the substrates were inoculated with 2% tertiary inoculum and incubated at room temperature (24–28°C) for 8 weeks. The tertiary inoculum refers to the pure culture of *Lentinula edodes* produced by inoculating the *Lentinula edodes* strain into Equation 1.

**Table 1:** Design table for different sawdust addition ratios.

**Treatment**	**Nutrient composition (%)**									
	**QS**	**MPP**	**VVP**	**ADP**	**ZJP**	**MAP**	**Bran**	**Sucrose**	**CaCO**_3_	**Gypsum**
T1 (CK)	80	-	-	-	-	-	17	1	1	1
T2	-	80	-	-	-	-	17	1	1	1
T3	40	40	-	-	-	-	17	1	1	1
T4	-	-	80	-	-	-	17	1	1	1
T5	40	-	40	-	-	-	17	1	1	1
T6	-	-	-	80	-	-	17	1	1	1
T7	40	-	-	40	-	-	17	1	1	1
T8	-	-	-	-	80	-	17	1	1	1
T9	40	-	-	-	40	-	17	1	1	1
T10	-	-	-	-	-	80	17	1	1	1
T11	40	-	-	-	-	40	17	1	1	1

QS, *Quercus* sawdust; MPP, *Malus pumila* pruning; VVP, *Vitis vinifera* pruning; ADP, *Actinidia deliciosa* purning; ZJP, *Ziziphus jujuba* pruning; MAP, *Morus alba* pruning.

T1 stand for QS; T2 stand for MPP; T3 stand for QS-MPP: 1:1; T4 stand for VVP; T5 stand for QS-VVP: 1:1; T6 stand for ADP; T7 stand for QS-ADP: 1:1; T8 stand for ZJP; T9 stand for QS-ZJP: 1:1; T10 stand for MAP; T11 stand for QS-MAP: 1:1.

Detachments, the practice of gently separating the substrate from bag walls without opening them, occurred every 15 days starting the first week after inoculation. To induce fruiting, the opened blocks were flushed, which involved rinsing with water to remove exudates. The blocks were then maintained in a climate-controlled chamber at 18 ± 1°C with 85–90% relative humidity. Following the first flush, blocks were submerged in water for 8 h to promote a second flush according to the methodology of Kobayashi et al. (27). Each flush lasted 20 days, with the blocks exposed to natural daylight during the day and kept in darkness at night.

### 2.2 Growth and production indicators

Mushroom growth was monitored daily, and mature fruiting bodies (white with upturned caps) were harvested by cutting the base just above the substrate using a sharp blade. Three harvest rounds were conducted for all substrate types throughout the experiment. During the growth process, several metrics were recorded: germination time measured as time from inoculation to the appearance of aerial hyphae, feeding time measured as time until hyphae extend into the medium, growth rate measured as daily mycelium extension, incubation period measured as time from inoculation to complete hyphae coverage, yield (measured as the fruiting body weight per kilogram of dry substrate), and pollution rate measured as percentage of contaminated bags.

### 2.3 Nutritional components and protein evaluation

The nutrient analysis of the raw material involved evaluation of the moisture content, carbohydrates, ash, crude protein, crude fat, fiber, and crude polysaccharides. These findings can indicate the nutrient richness of fruit tree branches as a measure of their potential suitability to serve as substrates for edible fungi cultivation.

#### 2.3.1 Moisture content

Moisture content determination was performed according to the Chinese standard GB 5009.3-2016 (National Food Safety Standard: Determination of Moisture in Foods) (28).

In brief, a flat aluminum was placed in a drying oven at 101–105°C for 1 h with the cap tilted and was then weighed after cooling for 30 min. This step was repeated until the weight stabilized within 2 mg. The sample was ground to <2 mm, and 2–10 mg of the sample was placed in the weighing bottle up to a thickness of 5 mm (10 mm for loose samples). The sample was dried in the oven at 101–105°C for 2–4 h, cooled, and weighed; these steps were repeated until the weight difference was within 2 mg, indicating a constant weight. The moisture content (g/100 mg) of the sample (*X*_1_) was then calculated using the following formula:


(1) $${\text{X}}_{1}\;=\;\frac{{\text{m}}_{1}\;-{\text{m}}_{2}}{{\text{m}}_{1}-{\text{m}}_{3}}\times 100$$

where *m*_1_ is the weight of the bottle (with sand and a glass rod) plus the sample (g), *m*_2_ is the weight after drying (g), and *m*_3_ is the weight of the bottle (with sand and the glass rod) alone (g).

#### 2.3.2 Ash content

Ash content determination was based on the Chinese standard GB 5009.4-2016 (National Food Safety Standard: Determination of Ash content in Foods) (29).

After weighing the sample, 1 mL of 240 g/L magnesium acetate solution was added to fully wet the sample. After 10 min, the sample was dried in a water bath and then heated gently on an electric plate until fully carbonized. The sample was then burned in a high-temperature furnace at 550 ± 25°C for 4 h to obtain the ash. After cooling to ~200°C, the ash was placed on a desiccator for 30 min before weighing. If carbon particles were present, the sample was moistened and redried as above until no carbon remained. The ashing process was repeated until consecutive weight measurements differed by no more than 0.5 mg. The ash content (g/100 g) in the sample (*X*_2_) was then calculated using the following formula:


(2) $${\text{X}}_{2}=\frac{{\text{m}}_{1}-{\text{m}}_{2}\;-\;{\text{m}}_{0\;}}{{\text{m}}_{3}-\;{\text{m}}_{2}}\times 100$$

where *m*_1_ is the weight of the crucible plus ash (g), *m*_2_ is the weight of the crucible alone (g), *m*_0_ is the weight of magnesium oxide (g), and *m*_3_ is the weight of the crucible plus sample (g).

#### 2.3.3 Crude protein content

Crude protein content determination was based on the Chinese standard GB 5009.5-2016 (National Food Safety Standard: Determination of Protein in Foods) (30).

In brief, 0.2 g of thoroughly mixed solid samples, 2 g of semi-solid samples, or 10 g of liquid samples was weighed to an accuracy of 0.001 g and placed in a digestion tube to which 0.4 g of copper sulfate, 6 g of potassium sulfate, and 20 mL of sulfuric acid were added. The mixture was digested in a digestion furnace. Once the furnace temperature reached 420°C, the digestion was continued for 1 h. Once the liquid in the digestion tube became green and transparent, the tube was removed from the furnace, cooled, and mixed with 50 mL of water. Automatic liquid addition, distillation, titration, and recording of titration data were performed with an automatic Kjeldahl nitrogen analyzer. The protein content (g/100 g) in the sample (*X*_3_) was then calculated with the following formula:


(3) $${\text{X}}_{3}\;=\;\frac{\left({\text{V}}_{1}-{\text{V}}_{2}\right)\times \text{c}\times 0.0140\;}{\text{m}\times {\text{V}}_{3}/100}\times \text{F}\times 100$$

Where *V*_1_ is the volume of standard sulfuric acid or hydrochloric acid solution used by the sample solution (mL); *V*_2_ is the volume used by the reagent blank (mL); *c* is the concentration of the standard acid solution (mol/L); *m* is the mass of the sample (g); *V*_3_ is the volume of the digest solution absorbed (mL); and *F* is the conversion factor for nitrogen to protein, which is typically 6.25.

#### 2.3.4 Crude fat content

Crude fat content determination was based on the Chinese standard GB 5009.6-2016 (National Food Safety Standard: Determination of Fat in Foods) (31).

The mixed sample was weighed to 2–5 g (with accuracy to 0.001 g) and transferred into a filter paper cylinder that was placed in a Soxhlet extractor connected to a pre-weighed receiving flask. Anhydrous ether was added to the flask and heated in a water bath for continuous reflux extraction (6–8 times per hour) for 6–10 h. Extract completion was confirmed based on a lack of oil stains on a frosted glass rod. The solvent was removed from the flask and the remaining evaporate (1–2 mL) was placed in a water bath. The residue was dried at 100 ± 5°C for 1 h, cooled in a desiccator for 30 min, and weighed; the above steps were repeated until a constant weight was achieved. The fat content (g/100 g) in the sample (*X*_4_) was then calculated using the following formula:


(4) $$X_{4}\;=\;\frac{m_{1}\;-\;m_{0}}{m_{2}\;}\;\times \;100$$

where *m*_1_ is the weight of the receiver flask plus the fat (g), *m*_0_ is the weight of the empty receiver flask (g), and *m*_2_ is the weight of the sample (g).

#### 2.3.5 Crude fiber content

Crude fiber content determination was based on the Chinese standard GB 5009.10-2016 (Determination of Crude Fiber in Plant-based Foods) (32).

The crushed sample (20 g) was transferred to a 500-mL Erlenmeyer flask with 200 mL of boiling 1.25% sulfuric acid. The sample was boiled gently for 30 min with shaking every 5 min to maintain a constant volume. The sample was then filtered through a linen cloth and washed until the liquid became pH of 7. The residue was then washed with 200 mL of boiling 1.25% potassium hydroxide solution, boiled for 30 min, and filtered again. The obtained residue was transferred to a pre-dried and weighed G2 crucible and washed thoroughly with hot water, followed by a wash with ethanol and ether. The crude fiber content of the sample (*X*_5_) was then calculated with the following formula:


(5) $$X_{5}=\frac{G\text{​}}{m\;}\times 100\%$$

Where G is mass of the residue (or the mass lost during the high-temperature ashing process) in grams (g) and *m* is mass of the sample (g).

#### 2.3.6 Crude polysaccharides content

Crude polysaccharides content determination was based on the Chinese standard NY/T 1676-2023 (Determination of Crude Polysaccharides in Edible Mushrooms Using Spectrophotometry) (33).

The sample (0.2–0.5 g) was placed in a microwave digestion vessel with 20 mL of water, mixed thoroughly, and refrigerated overnight at 4°C. The solution was mixed again and microwave extraction was performed at 140°C for 2 h. The obtained extract was transferred to a 250-mL beaker, the vessel was washed with additional water, and the washings were combined. The beaker was heated on a hot plate until no liquid was moving, followed by the addition of 5 mL of water. After stirring the sample and cooling to room temperature, 75 mL of anhydrous ethanol was slowly added, stirred, and the sample was refrigerated at 4°C for 12 h. The mixture was centrifuged at 5,000 rpm for 10 min, the supernatant was discarded, and the precipitate was combined with the pooled washings. The mixture was stirred, heated, and transferred to a volumetric flask. After dilution and filtering, the first 10–15 mL of the filtrate was discarded and the remaining 20–30 mL was collected for measurement. The glucose standard curve was constructed using various volumes of glucose solution and measuring the absorbance at 490 nm after reacting with phenol and sulfuric acid. The crude polysaccharide content (g/100 mg; *X*_6_) was then calculated from the sample's absorbance value based on the following formula:


(6) $$X_{6}\;=\;\frac{m_{1}\times V_{1}\;}{m_{2}\times V_{2}}\times 0.9\times \frac{1}{10^{6}}\times 100$$

where *m*_1_ is the sugar content from the standard curve (μg), *V*_1_ is the dilution volume of the sample (mL), *V*_2_ is the volume of the sample solution used for colorimetric measurement (mL), and *m*_2_ is the sample mass (g).

#### 2.3.7 Mineral content

Calcium, iron, potassium, zinc, copper, sodium, magnesium, and manganese contents of the samples were measured according to the Chinese standard GB 5009.268-2016 (National Food Safety Standard: Determination of Multiple Elements in Food) (34).

In brief, 0.2 g of solid sample was placed in a microwave digestion vessel. The samples were preheated with ethanol or CO_2_; to remove these substances, 5 mL of nitric acid was added and left to react for 1 h or overnight. The sample was digested using standard microwave conditions, cooled, and then the vessel was opened to release the gas. After rinsing the lid, the mixture was heated or ultrasonically degassed and the volume was adjusted to 25 mL with water. The blank solution and sample solution were injected into the inductively coupled plasma mass spectrometer (ICP-MS) to measure the signal response values of the target element and the internal standard element. The concentration of the target element in the digestion solution was determined based on the standard curve. The element content (mg/kg or mg/L) in the sample (*X*_7_) was then calculated according to the following formula:


(7) $$X_{7}\;=\;\frac{\left(\rho -\rho_{0}\right)\times V\times f}{m\times 1,000}$$

Where ρ is the element's mass concentration in the sample solution (μg/L), ρ_0_ is the concentration in the blank solution (μg/L), *V* is the volume of the sample digestion solution (mL), *f* is the dilution factor, and *m* is the sample mass or volume (g or mL).

Selenium was tested according to the Chinese standard GB 5009.93-2017 (National Food Safety Standard: Determination of Selenium in Food) (35).

Similarly, 0.2 g of solid sample (weighed to an accuracy of 0.001 g) was placed in a digestion tube to which 10 mL of nitric acid and 2 mL of hydrogen peroxide were added. The sample was mixed and digested in a microwave system according to recommended conditions. After digestion, the digest was cooled and transferred to a flask containing glass beads and heated until nearly dry. Subsequently, 5 mL of 6 mol/L hydrochloric acid was added, and the sample was heated until turning clear with white smoke. After cooling, the sample was transferred to a 10-mL volumetric flask with 2.5 mL of 100 g/L potassium ferricyanide solution, diluted to the mark, and mixed. A reagent blank was established. Using hydrochloric acid (5+95) as the carrier and sodium borohydride (8 g/L) as the reductant, standard samples were introduced into the instrument to stabilize readings, followed by the measurement of selenium standards. The standard curve of concentration vs. fluorescence intensity was plotted and the fluorescence intensities of blank and sample solutions were measured for quantification. The selenium content (mg/kg or mg/L) in the sample (*X*_8_) was then measured according to the following formula:


(8) $$X_{8}\;=\;\frac{\left(\rho -\rho_{0}\right)\times V}{m\times 1,000}$$

where ρ is the selenium concentration in the sample solution (μg/L), ρ_0_ is the selenium concentration in the blank solution (μg/L), *V* is the total volume of the sample digestion liquid (mL), and *m* is the sample weight or volume (g or mL).

#### 2.3.8 Amino acid content

Amino acid content determination was based on the Chinese standard GB 5009.124-2016 (National Food Safety Standard: Determination of Amino Acids in Foods) (36).

Two grams of a ground sample was placed in a hydrolysis tube, mixed with an equal volume of hydrochloric acid, and adjusted to a volume of ~10 mL with 6 mol/L hydrochloric acid. Subsequently, three to four drops of phenol were added, and the tube was frozen for 3–5 min, evacuated under vacuum, filled with nitrogen, and sealed. The sample was then hydrolyzed at 110 ± 1°C for 22 h. The hydrolysate was cooled and filtered into a 50-mL volumetric flask while rinsing the tube with water. The sample was diluted to 50 mL and mixed. One milliliter of the filtrate was transferred to a test tube, concentrated under reduced pressure at 40–50°C, and the residue was dissolved in 1–2 mL of water. The sampled was dried again and evaporated to dryness. After adding 1–2 mL of pH 2.2 sodium citrate buffer, the sample was mixed, filtered through a 0.22-μm membrane, and amino acid contents were measured with an amino acid analyzer. The sample concentrations were compared to those of mixed amino acid standard solutions using the external standard method based on the peak areas. The amino acid content (g/100 g) in the sample (*X*_9_) was calculated using the following formula:


(9) $$X_{9}\;=\;\frac{c_{i}\times F\times V\times M\;}{m\times 10^{9}}\times 100$$

where *c*_*i*_ is the concentration in the sample solution (nmol/mL), *F* is the dilution factor, *V* is the volume of the hydrolysate transferred and diluted (mL), *M* is the molar mass of the amino acid (g/mol), and *m* is the sample mass (g).

Using the Food and Agriculture Organization (FAO)/World Health Organization (WHO) 1973 revised pattern for essential amino acids (EAAs) in ideal proteins, the amino acid score (AAS) and chemical score (CS) of the samples were calculated with the following formulas:


(10) $$\begin{array}{l} AAS=\;aa/AA\left(FAO/WHO\right) \end{array}$$

(11) $$\begin{array}{l} CS=\;aa/AA\left(Egg\right) \end{array}$$

where *aa* is the amino acid content of the test samples (mg/g), *AA* is the same amino acid content in standard mode of FAO/WHO (mg/g), and *AA* (Egg) is the content of the same amino acid in egg protein (mg/g).

The essential amino acid index (EAAI) was calculated as follows:


(12) $$\begin{array}{l} EAAI=(\frac{Thr\;t}{Thr\;s}\times \frac{Val\;t}{Val\;s}\times \frac{Iso\;t}{Iso\;s}\times \frac{Leu\;t}{Leu\;s}\times \frac{Lys\;t}{Lys\;s}\\ \;{\times \frac{Met+Cys\;t}{Met+Cys\;s}\times \frac{Tyr+Phe\;t}{Tyr+Phe\;s})}^{\frac{1}{7}} \end{array}$$

where *t* is the amino acid content of the test sample protein (mg/g) and *s* is the amino acid content of the standard protein (mg/g).

Finally, the biological value (BV) of the sample was calculated as follows:


(13) $$\begin{array}{l} BV=1.09\times \;EAAI-11.7 \end{array}$$

### 2.4 Data analysis

The data were subjected to significance analysis and principal component analysis (PCA) using SPSS 22.0 software.

## 3 Results

### 3.1 Nutrient composition of the raw materials

The basic nutrient composition varied among different sawdust samples derived from *Quercus* sp., *Malus pumila, Vitis vinifera, Actinidia deliciosa, Ziziphus jujuba*, and *Morus alba* pruning. The carbohydrates ranged from 77.3 to 84.8%. The ranges of the moisture, ash, crude protein, crude fat, crude fiber, and crude polysaccharides contents were 8.75–12.9, 2.7–4.2, 1.27–5.9, 0.4–0.8, 19.6–49.2, and 0.44–0.93%, respectively. These findings suggested that the pruning residues of these fruit trees are sufficiently rich in nutrients, making them suitable substrates for edible fungi cultivation (Table 2).

**Table 2:** Nutritional components in branches of fruit trees.

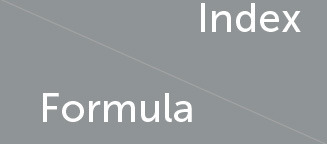	**Carbohydrates**	**Moisture**	**Ash**	**Crude protein**	**Crude fat**	**Coarse fiber**	**Crude polysaccharides**
QS	84.8 ± 0.02 ab	10.3 ± 0.07 d	3.1 ± 0.06 d	1.27 ± 0.08 e	0.5 ± 0.06 c	49.2 ± 0.04 a	0.65 ± 0.05 c
MPP	78.3 ± 0.09 c	8.75 ± 0.09 e	4.4 ± 0.03 a	7.79 ± 0.09 a	0.8 ± 0.02 a	27.3 ± 0.44 d	0.75 ± 0.07 b
VVP	77.3 ± 0.06 cd	13.5 ± 0.07 a	2.7 ± 0.09 e	5.9 ± 0.06 b	0.6 ± 0.15 b	44.6 ± 0.29 bc	0.67 ± 0.08 c
ADP	81.7 ± 0.08 b	11.1 ± 0.05 c	4.2 ± 0.05 ab	2.64 ± 0.05 d	0.4 ± 0.04 d	19.6 ± 0.21 e	0.65 ± 0.05 c
ZJP	81.5 ± 0.04 b	12.3 ± 0.06 ab	3.3 ± 0.09 c	3.3 ± 0.01 c	0.5 ± 0.11 c	43.2 ± 0.35 bc	0.93 ± 0.06 a
MAP	82.7 ± 0.03 a	12.9 ± 0.05 ab	2.7 ± 0.07 e	1.31 ± 0.03 e	0.4 ± 0.16 d	46.6 ± 0.17 b	0.44 ± 0.09 d

The data in the table are average and standard deviation (Mean ± SE). Multiple comparison was caried out by using Ducan method. The data with different small letters in column means significant difference (p <0.05). The data with same small letters in column means unsignificant different (P > 0.05).

### 3.2 Growth and production

The pruning residues of different fruit trees had significant impacts on the growth and development of *L. edodes* (Table 3). The germination stage ranged from 1.01 to 1.57 days, with *Ziziphus jujuba* pruning (ZJP) treatment resulting in the fastest germination. The feeding time ranged from 4.44 to 5.43 days, with *Actinidia deliciosa* pruning (ADP) treatment resulting in the quickest feeding time. The growth rate ranged from 2.58 to 3.64 mm/day, with the fastest growth rate observed for the *Quercus* sawdust (QS)-ZJP (1-1) group. The incubation period ranged from 30.14 to 38.42 days, with that of the QS-ZJP (1-1) group being the shortest. The yield ranged from 0.52 to 0.91 kg/kg, with the highest yield recorded in the QS-*Morus alba* pruning (MAP) (1-1) group. Compared to that of the control, growth was significantly promoted in the pruning substrate treatment groups, with QS-MAP (1-1) demonstrating the highest effectiveness in promoting growth, followed by QS-ZJP (1-1), QS-*Malus pumila* pruning (MPP) (1-1), QS-*Vitis vinifera* pruning (VVP) (1-1), MAP, and ZJP. It is important to note that the contamination rate of the MAP group reached 1.92%. Therefore, for future cultivation of *L. edodes* using MAP, it is recommended to add mepartricin to the substrate to reduce the potential of bacterial contamination.

**Table 3:** Effects of different fruit branches on growth and yield of *Lentinula edodes*.

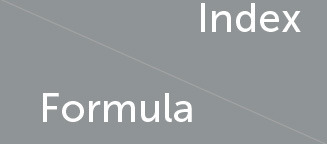	**Germination time/d**	**Feeding time/d**	**Growth rate/(mm/d)**	**Incubation period/d**	**Yield/(kg/kg)**	**Pollution rate/%**
CK	1.08 ± 0.54 g	4.70 ± 0.89 cd	3.05 ± 0.12 cd	35.17 ± 0.56 de	0.74 ± 0.01 ef	1.61 ± 0.04 d
T2	1.23 ± 0.29 cd	5.43 ± 0.92 a	3.07 ± 0.09 cd	38.12 ± 0.86 fg	0.59 ± 0.03 fg	0.98 ± 0.12 a
T3	1.26 ± 0.81 c	4.73 ± 0.35 cd	3.24 ± 0.08 b	34.25 ± 0.98 cd	0.85 ± 0.01 c	1.71 ± 0.19 d
T4	1.15 ± 0.21 f	5.10 ± 0.88 b	2.93 ± 0.05 de	42.15 ± 1.25 h	0.66 ± 0.02 h	1.83 ± 0.23 de
T5	1.37 ± 0.83 b	4.91 ± 0.70 bc	3.16 ± 0.05 bc	37.25 ± 0.87 fg	0.8 ± 0.03 d	1.32 ± 0.09 bc
T6	1.57 ± 0.72 a	4.44 ± 0.53 de	2.58 ± 0.05 f	48.52 ± 1.21 i	0.53 ± 0.01 i	1.91 ± 0.17 fg
T7	1.34 ± 0.29 bc	4.92 ± 0.72 bc	2.92 ± 0.04 de	38.42 ± 0.19 fg	0.7 ± 0.02 f g	1.81 ± 0.06 ef
T8	1.01 ± 0.19 hi	5.00 ± 0.06 bc	3.32 ± 0.02 b	32.09 ± 1.24 b	0.77 ± 0.03 e	1.22 ± 0.05 b
T9	1.06 ± 0.21 gh	4.70 ± 0.96 cd	3.64 ± 0.02 a	30.14 ± 1.41 a	0.88 ± 0.01 b	1.68 ± 0.17 d
T10	1.21 ± 0.35 de	4.92 ± 0.64 bc	3.18 ± 0.05 bc	33.09 ± 1.16 bc	0.78 ± 0.03 de	2.69 ± 0.11 h
T11	1.27 ± 0.62 c	5.10 ± 0.26 b	3.19 ± 0.04 bc	33.21 ± 0.48 bc	0.91 ± 0.02 a	1.92 ± 0.09 fg

The data in the table are average and standard deviation (Mean ± SE). Multiple comparison was caried out by using Ducan method. The data with different small letters in column means significant difference (p <0.05). The data with same small letters in column means unsignificant different (P > 0.05).

### 3.3 Routine nutrient analysis

Cultivating *L. edodes* on sawdust from the pruning residues of different fruit trees had a significant effect on the nutrient composition of its fruiting bodies (Table 4). The moisture content varied from 7.87 to 12.99%, with the MPP group having the lowest level. The ash content ranged from 5.42 to 6.57 g/100 g, with the highest level recorded in the MAP group. The crude protein level ranged between 24.86 and 30.16 g/100 g, peaking in the QS-MAP (1-1) group. The crude fat content was the highest in the ZJP group, ranging from 15.67 to 21.82%, while the crude fiber content ranged from 0.26 to 1.72 g/100 g, with the QS-MPP (1-1) group having the highest level. The crude polysaccharide content varied from 0.65 to 1.37 g/100 g, with the ZJP group showing the highest concentration. Notably, VVP, QS-ADP (1-1), MAP, and QS-MAP (1-1) significantly increased the levels of crude polysaccharides, crude fat, crude fiber, ash, and crude protein in *L. edodes* compared with those of the control.

**Table 4:** Effect of different fruit tree branches on routine nutrition components of *Lentinula edodes*.

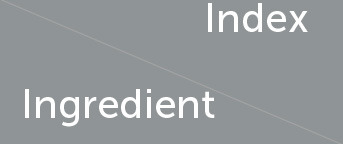	**Moisture content**	**Ash**	**Crude protein**	**Crude fat**	**Coarse fiber**	**Crude polysaccharides**
CK	9.22 ± 0.02 d	5.42 ± 0.01 bc	27.21 ± 0.02 c	19.81 ± 0.02 c	0.72 ± 0.07 e	0.65 ± 0.01 f
T2	7.93 ± 0.04 f	5.53 ± 0.02 bc	28.9 ± 0.05 b	17.92 ± 0.03 d	0.92 ± 0.06 cd	0.93 ± 0.03 d
T3	8.41 ± 0.03 e	5.72 ± 0.03 b	29.51 ± 0.02 b	15.67 ± 0.04 e	1.72 ± 0.04 a	1.28 ± 0.04 b
T4	11.5 ± 0.01 b	5.84 ± 0.08 b	27.85 ± 0.06 c	20.14 ± 0.06 ab	1.05 ± 0.08 bc	1.24 ± 0.01 b
T5	7.87 ± 0.04 f	5.52 ± 0.03 bc	29.11 ± 0.04 b	18.33 ± 0.05 d	1.02 ± 0.03 bc	1.07 ± 0.07 c
T6	12.99 ± 0.05 a	6.15 ± 0.06 ab	26.96 ± 0.02 cd	18.68 ± 0.08 d	0.26 ± 0.09 h	0.95 ± 0.02 d
T7	9.24 ± 0.01 d	5.59 ± 0.04 bc	27.41 ± 0.03 c	20.95 ± 0.07 ab	0.96 ± 0.01 cd	1.08 ± 0.03 c
T8	10.72 ± 0.02 c	5.76 ± 0.07 b	24.86 ± 0.09 e	21.82 ± 0.02 a	0.48 ± 0.02 g	1.37 ± 0.04 a
T9	10.06 ± 0.07 c	6.11 ± 0.09 ab	28.91 ± 0.06 b	19.37 ± 0.05 c	0.62 ± 0.05 f	1.03 ± 0.08 c
T10	10.86 ± 0.09 c	6.57 ± 0.11 a	29.75 ± 0.08 b	20.51 ± 0.04 ab	0.96 ± 0.03 cd	0.82 ± 0.07 e
T11	10.21 ± 0.02 c	6.32 ± 0.01 a	30.16 ± 0.09 a	19.36 ± 0.01 c	1.19 ± 0.06 b	0.93 ± 0.04 d

The data in the table are average and standard deviation (Mean ± SE). Multiple comparison was caried out by using Ducan method. The data with different small letters in column means significant difference (p <0.05). The data with same small letters in column means unsignificant different (P > 0.05).

### 3.4 Mineral element analysis

The mineral content in *L. edodes* showed notable variations across the six different substrates (Table 5). MPP resulted in the highest calcium content at 250 mg/kg, which was 37.6% higher than that of the control. *Vitis vinifera, Ziziphus jujuba*, and *Morus alba* pruning promoted calcium accumulation. QS-MPP (1-1) as a substrate led to the highest potassium content of *L. edodes* at 3.10 × 10^4^ mg/kg, which was 23.9% above that of the control. The highest iron content was observed in the VVP group, at 92.1 mg/kg, which was 64.2% higher than that of the control. VVP also resulted in the highest copper content at 6.71 mg/kg, which was 39.5% higher than that of the control. The sodium content was the highest in the MPP group at 124.1 mg/kg, which was 40.5% higher than that of the control. Treatment groups with fruit tree pruning residues added as the cultivation substrate showed increased levels of potassium, iron, copper, and sodium compared to those of the control, suggesting that sawdust from these five types of fruit tree branches enhanced the accumulation of these minerals.

**Table 5:** Effects of different fruit branches on mineral elements of *Lentinula edodes*.

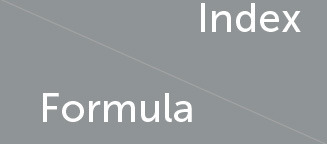	**Calcium**	**Potassium**	**Iron**	**Zinc**	**Selenium**	**Copper**	**Sodium**	**Manganese**	**Magnesium**
CK	156 ± 1.23 g	2.36 × 10^4^ ± 0.04 × 10^4^ g	33.0 ± 1.36 f	53.4 ± 1.24 c	0.504 ± 0.03 i	4.06 ± 1.21 c	73.8 ± 3.52 g	15.7 ± 0.23 bc	1.22 × 10^3^ ± 0.09 × 10^4^ e
T2	217 ± 0.97 b	2.72 × 10^4^ ± 0.36 × 10^4^ e	65.4 ± 0.93 b	72.6 ± 0.95 b	0.967 ± 0.12 g	5.68 ± 0.94 ab	124 ± 2.93 a	16.8 ± 0.21 b	1.48 × 10^3^ ± 0.01 × 10^4^ b
T3	250 ± 0.24 a	3.10 × 10^4^ ± 0.12 × 10^4^ a	63.2 ± 0.92 b	76.4 ± 1.46 b	0.739 ± 0.04 h	6.06 ± 0.19 a	99.6 ± 1.37 c	20.4 ± 0.19 a	1.64 × 10^3^ ± 0.04 × 10^4^ d
T4	143 ± 1.42 h	2.81 × 10^4^ ± 0.09 × 10^4^ d	92.1 ± 1.21 a	86.6 ± 0.46 a	1.38 ± 0.28 d	6.71 ± 0.72 a	95.6 ± 0.99 cd	12.4 ± 0.28 d	1.22 × 10^3^ ± 0.05 × 10^4^ e
T5	210 ± 1.86 c	2.69 × 10^4^ ± 0.14 × 10^4^ ef	60.4 ± 2.56 b	88.7 ± 1.63 a	0.527 ± 0.03 i	5.6 ± 0.35 ab	99 ± 3.72 c	16.4 ± 0.63 b	1.40 × 10^3^ ± 0.04 × 10^4^ bc
T6	130 ± 0.92 I	2.78 × 10^4^ ± 0.06 × 10^4^ de	60.8 ± 0.96 b	75.9 ± 0.97 b	1.54 ± 0.12 c	5.46 ± 0.29 ab	91.5 ± 4.15 cd	11.8 ± 1.02 de	1.34 × 10^3^ ± 0.03 × 10^4^ cd
T7	144 ± 1.73 h	2.91 × 10^4^ ± 0.21 × 10^4^ Cc	35.2 ± 4.24 f	77.3 ± 1.52 b	1.23 ± 0.03 f	5.75 ± 0.48 ab	87.8 ± 2.18 e	12.9 ± 0.9 d	1.40 × 10^3^ ± 0.11 × 10^4^ bc
T8	158 ± 2.19 g	2.54 × 10^4^ ± 0.04 × 10^4^ f	45.4 ± 2.16 e	50.5 ± 1.78 c	4.58 ± 0.27 a	4.68 ± 0.62 c	83 ± 2.84 f	12.4 ± 0.04 d	1.18 × 10^3^ ± 0.01 × 10^4^ e
T9	198 ± 2.55 cd	2.98 × 10^4^ ± 0.12 × 10^4^ b	52.4 ± 1.72 cd	69.6 ± 0.92 b	2.16 ± 0.97 b	6.1 ± 0.26 a	90.3 ± 1.65 cd	16.2 ± 0.09 b	1.42 × 10^3^ ± 0.09 × 10^4^ bc
T10	168 ± 3.82 f	2.59 × 10^4^ ± 0.08 × 10^4^ f	59.2 ± 1.36 c	70.2 ± 1.58 b	1.36 ± 0.46 e	5.92 ± 0.52 a	102.8 ± 2.25 b	14.7 ± 0.11 c	1.26 × 10^3^ ± 0.04 × 10^4^ e
T11	175 ± 0.97 e	2.82 × 10^4^ ± 0.05 × 10^4^ d	64.9 ± 4.21 b	75.5 ± 2.64 b	1.29 ± 0.72 f	5.19 ± 0.47 bc	98.4 ± 1.56 bc	16.8 ± 0.31 b	1.37 × 10^3^ ± 0.05 × 10^4^ cd

The data in the table are average and standard deviation (Mean ± SE). Multiple comparison was caried out by using Ducan method. The data with different small letters in column means significant difference (p <0.05). The data with same small letters in column means unsignificant different (P > 0.05).

The highest manganese content was found in the QS-MPP (1-1) treatment group at 16.8 mg/kg, which was 6.5% higher than that of the control. QS-MPP (1-1) also resulted in the highest magnesium content at 1.64 × 10^3^ mg/kg, which was 25.6% above that of the control. The mixtures of QS-ZJP and QS-MAP increased both magnesium and manganese levels, whereas *Vitis vinifera* and *Actinidia deliciosa* pruning residues did not lead to manganese accumulation. The highest zinc content was observed in the QS-VVP (1-1) mixture group at 88.7 mg/kg, which was 39.9% higher than that of the control. ZJP resulted in the highest selenium content at 4.58 mg/kg, which was 88.9% higher than that of the control. MAP, sourced from selenium-rich regions, exhibited a high selenium content in the mushroom.

### 3.5 Amino acid nutritional value

#### 3.5.1 Composition of amino acids

The substrates derived from various fruit tree branches contained 17 amino acids at different concentrations (Table 6). In the ZJP and QS-MAP (1-1) groups, the proportion of EAAs to total amino acids (TAAs) was 34.34 and 34.28%, respectively, which was 0.34 and 0.28% higher than that of the control. The EAA content in the QS-MPP (1-1) and QS-VVP (1-1) groups was 7.73 and 7.58%, respectively, which was 0.26 and 0.11% higher than that of the control. The QS-MPP (1-1), QS-VVP (1-1), and QS-ADP (1-1) groups had non-essential amino acid (NEAA) contents of 15.26, 15.66, and 14.77%, respectively, which were 0.76, 1.16, and 0.25% higher than those of the control. Glutamic acid had the highest content among all amino acids, whereas cysteine was found at the lowest level. The EAA/TAA ratio ranged from 32.53 to 34.34% and the EAA/NEAA ratio ranged from 48.21 to 52.29%.

**Table 6:** Effects of different fruit branches on amino acid composition of *Lentinula edodes*.

**AA classification**	**Type of amino acid**	**Formula**										
		**CK**	**T2**	**T3**	**T4**	**T5**	**T6**	**T7**	**T8**	**T9**	**T10**	**T11**
Essential amino acid	Threonine	1.08 ± 0.002 ab	1.02 ± 0.013 bc	1.12 ± 0.001 a	1.04 ± 0.004 ab	1.11 ± 0.012 a	0.99 ± 0.002 c	1.08 ± 0.004 ab	1.01 ± 0.009 bc	1.08 ± 0.008 ab	1.05 ± 0.009 bc	1.12 ± 0.003 a
	Valine	1.25 ± 0.011 ab	1.17 ± 0.015 cd	1.29 ± 0.012 a	1.17 ± 0.009 cd	1.29 ± 0.017 a	1.14 ± 0.007 cd	1.18 ± 0.017 cd	1.1 ± 0.012 de	1.21 ± 0.009 bc	1.24 ± 0.024 ab	1.28 ± 0.036 a
	Isoleucine	1.26 ± 0.025 b	1.24 ± 0.016 b	1.34 ± 0.067 a	1.08 ± 0.021 cd	1.17 ± 0.052 bc	1.15 ± 0.019 bc	1.14 ± 0.026 bc	1.24 ± 0.026 b	1.28 ± 0.038 ab	1.19 ± 0.029 bc	1.21 ± 0.019 b
	Methionine	0.44 ± 0.015 a	0.39 ± 0.011 ab	0.42 ± 0.009 a	0.37 ± 0.004 ab	0.41 ± 0.002 a	0.4 ± 0.001 a	0.37 ± 0.004 ab	0.38 ± 0.012 ab	0.43 ± 0.016 a	0.35 ± 0.018 ab	0.42 ± 0.024 a
	Leucine	1.42 ± 0.001 b	1.38 ± 0.016 bc	1.47 ± 0.019 b	1.36 ± 0.023 bc	1.51 ± 0.028 a	1.32 ± 0.035 bc	1.38 ± 0.024 bc	1.25 ± 0.027 d	1.39 ± 0.018 bc	1.36 ± 0.032 bc	1.39 ± 0.019 bc
	Phenylalanine	1.05 ± 0.025 ab	0.99 ± 0.052 bc	1.07 ± 0.062 a	0.93 ± 0.018 bc	1.07 ± 0.032 a	0.94 ± 0.054 bc	0.98 ± 0.027 bc	0.89 ± 0.073 cd	0.99 ± 0.027 bc	1.02 ± 0.083 ab	1.05 ± 0.023 ab
	Lysine	0.97 ± 0.019 ab	0.95 ± 0.061 ab	1.02 ± 0.041 a	0.97 ± 0.069 ab	1.02 ± 0.017 a	0.94 ± 0.072 ab	0.99 ± 0.036 ab	0.87 ± 0.047 bc	0.96 ± 0.049 ab	0.93 ± 0.027 ab	0.92 ± 0.063 ab
Noessential amino acid	Aspartate	1.97 ± 0.014 ab	1.92 ± 0.027 ab	2.06 ± 0.038 a	1.9 ± 0.019 ab	2.09 ± 0.052 a	1.83 ± 0.016 bc	1.95 ± 0.052 ab	1.81 ± 0.018 bc	1.93 ± 0.019 ab	1.95 ± 0.036 ab	2.01 ± 0.027 a
	Serine	1.04 ± 0.016 a	1.01 ± 0.051 a	1.1 ± 0.013 a	1.01 ± 0.015 a	1.08 ± 0.024 a	0.96 ± 0.029 ab	1.05 ± 0.046 a	0.99 ± 0.024 ab	1.05 ± 0.015 a	0.95 ± 0.026 ab	1.02 ± 0.063 a
	Cysteine	0.11 ± 0.015 a	0.13 ± 0.063 a	0.15 ± 0.027 a	0.13 ± 0.028 a	0.14 ± 0.039 a	0.12 ± 0.017 a	0.16 ± 0.003 a	0.14 ± 0.013 a	0.13 ± 0.017 a	0.14 ± 0.021 a	0.12 ± 0.042 a
	Glutamate	6.31 ± 0.072 ab	6.0 ± 0.092 bc	6.6 ± 0.018 ab	6.05 ± 0.025 bc	6.91 ± 0.036 a	5.62 ± 0.032 cd	6.38 ± 0.057 ab	5.34 ± 0.028 cd	6.15 ± 0.024 ab	6.05 ± 0.036 bc	5.89 ± 0.025 cd
	Glycine	0.99 ± 0.062 ab	0.96 ± 0.015 ab	1.03 ± 0.063 a	0.93 ± 0.084 ab	1.02 ± 0.027 a	0.91 ± 0.038 ab	0.93 ± 0.084 ab	0.89 ± 0.038 ab	0.96 ± 0.036 ab	0.94 ± 0.012 ab	0.92 ± 0.017 ab
	Alanine	1.03 ± 0.016 ab	1.05 ± 0.012 ab	1.1 ± 0.061 a	1.02 ± 0.081 ab	1.13 ± 0.019 a	1.14 ± 0.016 a	1.18 ± 0.063 a	0.95 ± 0.058 ab	1.01 ± 0.048 ab	1.02 ± 0.027 ab	1.09 ± 0.083 ab
	Tyrosine	0.18 ± 0.017 c	0.25 ± 0.013 ab	0.26 ± 0.061 ab	0.29 ± 0.081 a	0.29 ± 0.028 a	0.26 ± 0.083 a	0.3 ± 0.025 a	0.25 ± 0.016 ab	0.27 ± 0.037 a	0.23 ± 0.083 ab	0.27 ± 0.024 a
	Histidine	0.42 ± 0.016 a	0.41 ± 0.012 a	0.45 ± 0.019 a	0.4 ± 0.012 a	0.44 ± 0.031 a	0.39 ± 0.021 ab	0.42 ± 0.018 a	0.36 ± 0.037 ab	0.4 ± 0.029 a	0.41 ± 0.016 a	0.39 ± 0.026 ab
	Arginine	1.38 ± 0.037 c	1.34 ± 0.036 c	1.45 ± 0.029 ab	1.38 ± 0.024 c	1.50 ± 0.026 a	1.22 ± 0.084 d	1.37 ± 0.027 c	1.16 ± 0.035 d	1.31 ± 0.033 c	1.32 ± 0.043 c	1.41 ± 0.018 ab
	Proline	1.07 ± 0.016 a	1.01 ± 0.013 ab	1.06 ± 0.018 ab	1.01 ± 0.013 ab	1.06 ± 0.042 ab	1.02 ± 0.052 ab	1.03 ± 0.026 ab	1.0 ± 0.019 ab	1.09 ± 0.027 a	1.02 ± 0.043 ab	1.05 ± 0.026 ab
Total essential amino acid		7.47 ± 0.128 bc	7.14 ± 0.262 d	7.73 ± 0.262 a	6.92 ± 0.182 e	7.58 ± 0.236 b	6.88 ± 0.235 ef	7.12 ± 0.236 d	6.74 ± 0.244 g	7.34 ± 0.199 bc	7.14 ± 0.284 d	7.39 ± 0.277 bc
Total noessential amino acid		14.5 ± 0.241 b	14.08 ± 0.256 b	15.26 ± 0.296 a	14.12 ± 0.348 b	15.66 ± 0.248 a	13.47 ± 0.343 c	14.77 ± 0.303 b	12.89 ± 0.244	14.3 ± 0.251 b	14.03 ± 0.281 b	14.17 ± 0.261 b
Total amino acids		21.97 ± 0.369 ab	21.22 ± 0.581 ab	22.99 ± 0.558 a	21.04 ± 0.530 ab	23.24 ± 0.484 a	20.35 ± 0.578 bc	21.89 ± 0.539 ab	19.63 ± 0.488 bc	21.64 ± 0.45 ab	21.17 ± 0.565 ab	21.56 ± 0.538 ab
EAA/TAA		34.00 a	33.65 ab	33.62 ab	32.89 bc	32.62 bc	33.81 ab	32.53 bc	34.34 a	33.92 ab	33.73 bc	34.28 a
EAA/NEAA		51.52 b	50.71 bc	50.66 bc	49.01 d	48.4 e	51.08 b	48.21 e	52.29 a	51.33 b	50.89 bc	52.15 a

The data in the table are average and standard deviation (Mean ± SE). Multiple comparison was caried out by using Ducan method. The data with different small letters in column means significant difference (p <0.05). The data with same small letters in column means unsignificant different (P > 0.05).

The levels of valine, isoleucine, and threonine in all tested samples exceeded the corresponding values in the FAO/WHO model, whereas the levels of leucine, tyrosine-phenylalanine (Tyr-Phe), lysine, and methionine-cysteine (Met-Cys) were lower than those of the FAO/WHO model (Table 7). The contents of valine, isoleucine, and threonine in *L. edodes* cultivated on the six different substrates surpassed those in the FAO/WHO model. Notably, the Tyr-Phe content in the QS-MAP (1-1) group was higher than that in the FAO/WHO model. When compared to the whole-egg model, only the ZJP and QS-MAP (1-1) groups had equivalent threonine levels, with the QS-MAP (1-1) group showing an 11.95% higher threonine content than that of the whole-egg model. *L. edodes* is a high-quality amino acid source and thus its regular consumption is recommended for health benefits. Despite its high amino acid content, there remains a gap compared to standard protein sources, indicating its role as a supplementary protein source.

**Table 7:** Comparison of essential amino acids and amino acid patterns.

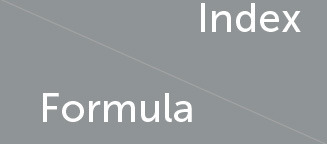	**Val**	**Iso**	**Leu**	**Lys**	**Met+Cys**	**Tyr+Phe**	**Thr**
CK	56.9	57.35	64.64	44.15	25.03	55.99	49.16
T2	55.14	58.44	65.03	44.77	24.51	58.44	48.07
T3	56.11	58.29	63.94	44.37	24.79	57.85	48.72
T4	55.61	51.33	64.64	46.1	23.77	57.99	49.43
T5	55.51	50.34	64.97	43.89	23.67	58.52	47.76
T6	56.02	56.51	64.87	46.19	25.55	58.97	48.65
T7	53.91	52.08	63.04	45.23	24.21	58.47	49.34
T8	56.04	63.17	63.68	44.32	26.49	58.07	51.45
T9	55.92	59.15	64.23	44.36	25.88	58.23	49.91
T10	58.57	56.21	64.24	43.93	23.15	59.05	49.6
T11	59.37	56.12	64.47	42.67	25.05	61.22	51.95
FAO/WHO model	50	40	70	55	35	60	40
All-egg model	73	66	88	64	55	100	51

#### 3.5.2 Flavor amino acids

The amino acid content was ranked in the samples from the highest to lowest as follows: medicinal amino acids (MAAs), umami amino acids (UAAs), bitter-tasting amino acids (BAAs), and sweet-tasting amino acids (SAAs). Glutamic acid was the most abundant and predominant UAA in all formulations. The proportion of UAAs to TAAs was notably high, exceeding 35%, with the highest level of 38.73% observed in the QS-VVP (1-1) group. The SAA content ranged from 18.56 to 19.80%, with the ADP group showing the highest percentage. The BAA content varied from 32.53 to 34.34%, with the ZJP group having the highest amount. UAA levels were 1.621–1.758 times >BAA levels, indicating relatively high proportions of UAAs and SAAs than other amino acid types, which can contribute to a better taste and thus offer greater development potential. The highest proportions of UAAs, SAAs, and BAAs were found in the QS-VVP (1-1), ADP, and ZJP groups, respectively, underscoring their significance in flavor profile development (Table 8).

**Table 8:** Effects of different fruit branches on flavor amino acids of *Lentinula edodes*.

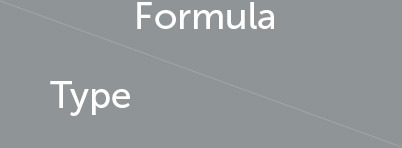	**CK**	**T2**	**T3**	**T4**	**T5**	**T6**	**T7**	**T8**	**T9**	**T10**	**T11**
MAA	14.7 b	14.18 bc	15.38 a	14.18 bc	15.82 a	13.44 d	14.65 b	12.84 d	14.39 bc	14.15 bc	14.28 bc
UAA	8.28 ab	7.92 ab	8.66 ab	7.95 ab	9.0 a	7.45 bc	8.33 ab	7.1 bc	8.08 ab	8.0 ab	7.9 ab
SAA	4.13 ab	4.03 ab	4.29 a	3.97 bc	4.29 a	4.03 ab	4.19 ab	3.83 bc	4.11 ab	3.93 bc	4.08 ab
BAA	7.47 a	7.14 b	7.73 a	6.92 c	7.58 a	6.88 c	7.12 b	6.74 c	7.34 a	7.14 b	7.39 a
UAA/TAA (%)	37.69 ab	37.32 ab	37.67 ab	37.79 ab	38.73 a	36.61 bc	38.05 a	36.42 bc	37.34 ab	37.79 ab	36.64 bc
SAA/TAA (%)	18.80 ab	18.99 ab	18.66 bc	18.87 ab	18.46 bc	19.80 a	19.14 ab	19.51 a	18.99 ab	18.56 bc	18.92 ab
BAA/TAA (%)	34.00 a	33.65 ab	33.62 ab	32.89 bc	32.62 bc	33.81 ab	32.53 bc	34.34 a	33.92 ab	33.73 ab	34.28 a
(UAA+SAA)/BAA (%)	1.661 ab	1.674 ab	1.675 ab	1.723 a	1.753 a	1.669 ab	1.758 a	1.629 ab	1.661 ab	1.671 ab	1.621 ab

MAA crucial for maintaining body nitrogen balance, include Glu, Asp, Arg, Gly, Phe, Tyr, Met, Leu, and Lys. UAA consist of Glu and Asp, while SAA include Ser, Gly, Ala, and Pro. BAA comprise essential amino acids.

#### 3.5.3 Protein quality

Both protein quality assessment scores highlighted amino acid imbalances in various formulations of *L. edodes*, with consistently identified deficiencies (Table 9). According to the AAS, Met-Cys was the primary limiting amino acid across all treatments, ranging from 66.1 to 75.7%, while lysine was the second most limiting amino acid, ranging from 77.6 to 84%. The CS results corroborated the AAS findings, showing that the first and second limiting amino acids ranged from 42.1 to 48.2% and from 66.7 to 72.2%, respectively.

**Table 9:** AAS, CS, EAAI, and BV of amino acids in *Lentinula edodes*.

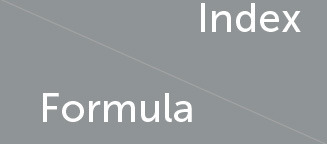	**Val**		**Iso**		**Leu**		**Lys**		**Met**+ **Cys**		**Tyr**+ **Phe**		**Thr**		**EAAI**		**BV**	
	**AAS**	**CS**	**AAS**	**CS**	**AAS**	**CS**	**AAS**	**CS**	**AAS**	**CS**	**AAS**	**CS**	**AAS**	**CS**	**AAS**	**CS**	**AAS**	**CS**
CK	1.138	0.779	1.434	0.869	0.923	0.735	0.803	0.999	0.715	0.455	0.933	0.560	1.229	0.964	99.88	70.23	97.17	64.85
T2	1.103	0.755	1.461	0.885	0.929	0.739	0.814	1.000	0.700	0.446	0.974	0.584	1.202	0.943	99.98	70.30	97.28	64.93
T3	1.122	0.769	1.458	0.883	0.913	0.727	0.807	100.03	0.708	0.451	0.964	0.579	1.218	0.955	100.03	70.34	97.34	64.97
T4	1.112	0.762	1.283	0.778	0.923	0.735	0.838	98.44	0.679	0.432	0.967	0.580	1.236	0.969	98.44	69.22	95.60	63.75
T5	1.110	0.760	1.259	0.763	0.928	0.738	0.798	97.12	0.676	0.430	0.975	0.585	1.194	0.936	97.12	68.29	94.16	62.74
T6	1.120	0.767	1.413	0.856	0.927	0.737	0.840	101.04	0.730	0.465	0.983	0.590	1.216	0.954	101.04	71.04	98.43	65.74
T7	1.078	0.738	1.302	0.789	0.901	0.716	0.822	97.94	0.692	0.440	0.975	0.585	1.234	0.967	97.94	68.87	95.06	63.36
T8	1.121	0.768	1.579	0.957	0.910	0.724	0.806	102.91	0.757	0.482	0.968	0.581	1.286	1.009	102.91	72.36	100.47	67.17
T9	1.118	0.766	1.479	0.896	0.918	0.730	0.807	101.32	0.739	0.471	0.971	0.582	1.248	0.979	101.32	71.24	98.74	65.95
T10	1.171	0.802	1.405	0.852	0.918	0.730	0.799	99.62	0.661	0.421	0.984	0.591	1.240	0.973	99.62	70.05	96.89	64.65
T11	1.187	0.813	1.403	0.850	0.921	0.733	0.776	101.75	0.716	0.455	1.020	0.612	1.299	1.019	101.75	71.54	99.21	66.28

The EAAI of *L. edodes* cultivated on sawdust from fruit tree branches ranged from 97.12 to 102.91, closely matching the FAO/WHO standard amino acid model. The QS-MAP (1-1), QS-ZJP (1-1), ADP, and QS-MPP (1-1) groups all had EAAI values exceeding 100, indicating a well-rounded nutritional profile. According to the whole-egg model, the EAAI ranged from 68.29 to 72.36, with the ZJP group showing the highest value at 72.36, followed by the QS-MAP (1-1) group at 71.54. This suggests that incorporating *Morus alba* and *Ziziphus jujuba* pruning residues enhances the nutritional quality of *L. edodes*.

The BV of the protein, which measures the efficiency of protein utilization after digestion, ranged from 94.16 to 100.47% based on FAO/WHO standards, and from 62.74 to 67.17% when compared to the whole-egg model. A higher BV indicates superior nutritional quality. The ZJP group had the highest BV of 100.47%, suggesting that using *Morus alba* pruning residue as a substrate can significantly enhance the protein content of *L. edodes*.

### 3.6 PCA

To more comprehensively evaluate the impact of varying proportions of fruit tree pruning residues on the growth and development of *L. edodes*, several indicators were analyzed together in a multivariate analysis with PCA, including mycelial growth rate, yield, biological efficiency, crude protein, calcium, BAA, TAA, UAA, SAA, MAA, and TAA. Prior to conducting PCA, data suitability was confirmed through the Kaiser–Meyer-Olkin and Bartlett tests, which validated the use of this method.

The eigenvalues of the first two principal components both exceeded 1, with variance explained ratios of 64.09 and 21.83%, respectively. The cumulative variance explained reached 85.92%, demonstrating that the first two principal components effectively captured the differences across group indicators. PC1 primarily represented the variation in factors such as BAA, TAA, UAA, SAA, MAA (medicinal amino acids), and crude protein, with BAA having the strongest positive influence on this component. PC2 mainly captured the variation in growth rate, biological efficiency, yield, and calcium, with growth rate exerting the strongest positive influence on this component (Table 10).

**Table 10:** Short array of component loadings after principal component analysis rotation.

**Index**	**PC1**	**PC2**
Mycelial growth rate	0.19	0.5514
Yield	0.2669	0.4357
Biological efficiency	0.2829	0.4563
Crude protein	0.2849	0.0115
Ca	0.3049	0.1197
TAA	0.3708	−0.2222
TMAA	0.3595	−0.2557
UAA	0.3507	−0.261
SAA	0.3252	−0.3112
BAA	0.3782	−0.0605
Eigenvalues	6.409	2.183
Variance explained ratio/%	64.093	21.827
Cumulative explained ratio/%	64.093	85.92

By combining the variance explained by the first two principal components and applying the proportion of each component's eigenvalue to the total eigenvalue of the extracted components as weights, the integrated evaluation model was derived as follows:


(14) $$\begin{array}{l} Y=0.746\times X_{1}+0.254\times X_{2} \end{array}$$

where *X*_1_ is PC1 and *X*_2_ is PC2.

Based on the constructed model, the comprehensive scores of *L. edodes* under each treatment were calculated (Table 11). The QS-MPP (1-1) treatment achieved the highest score, indicating that using *Malus pumila* pruning yields the best overall results. This was closely followed by QS-VVP (1-1), QS-ZJP (1-1), QS-MAP (1-1), and QS, showing improved quality when *L. edodes* is cultivated with *Vitis vinifera, Morus alba, Ziziphus jujuba* pruning, and *Quercus* sawdust, respectively. The remaining treatments had negative comprehensive scores, indicating relatively lower yields and quality of *L. edodes* cultivated with these formulations.

**Table 11:** Composite scores and ranking of *Lentinula edodes* under different treatment.

**Treatment**	**PC1**	**PC2**	**Composite score**	**Ranking**
T1	0.4282	−0.7445	0.1303	5
T2	−0.5407	−0.3803	−0.4999	7
T3	3.8749	−0.2088	2.8375	1
T4	−1.8748	−0.69398	−1.5748	9
T5	3.5083	−1.2304	2.3045	2
T6	−3.8991	−2.0552	−3.4307	11
T7	−0.2403	−1.4276	−0.5419	8
T8	−3.6799	2.4485	−2.1230	10
T9	1.5443	2.0023	1.6606	3
T10	−0.3367	0.8273	−0.0409	6
T11	1.2158	1.4626	1.2785	4

## 4 Discussion

Orchard waste has the potential to be used as edible mushroom cultivation material. Through our experiments, we found that *Malus pumila, Vitis vinifer*a, *Ziziphus jujuba*, and *Morus alba* pruning residues can be utilized for *L. edodes* cultivation. The use of sawdust from fruit tree branches for cultivating *L. edodes* is a viable option. Both pure sawdust from the fruit tree branches and partially mixed sawdust can be utilized for *L. edodes* cultivation, although the ratios of these additions significantly impact mycelial growth and fruit body development (Tables 3, 4). According to data released by the National Bureau of Statistics, in 2020, the fruit orchard area in China reached 1.265 million hectares (37, 38). With the continuous increase in fruit planting areas, the amount of waste branches generated during the daily management of orchards has also risen (26). Currently, most fruit tree pruning residues are treated as waste, either being removed from orchards or used as firewood. This practice not only wastes resources but also causes environmental pollution. Research indicates that fruit tree branches discarded as waste are rich in organic matter, mineral components, and cellulose, with the organic matter content exceeding 95% and the carbon content exceeding 45%, which highlights their significant resource utilization potential (39, 40). The nutrient profiles of substrates directly influence the nutritional content of *L. edodes* fruiting bodies. Substrates with higher ash, crude protein, and crude fat contents, such as those from *Morus alba* and *Quercus*, produced fruiting bodies with higher levels of these nutrients. Substrates with higher moisture content and specific nutrient profiles, such as those from *Malus pumila* and *Ziziphus jujuba*, resulted in different nutrient concentrations in the fruiting bodies. Optimizing substrate composition is crucial for enhancing the nutritional quality and yield of *L. edodes*. This study provides a scientific basis for utilizing fruit tree pruning residues in *L. edodes* cultivation, supporting the appropriate utilization of these available resources.

This study evaluated the impact of various fruit tree pruning residues on the growth and yield of *L. edodes*. QS-ZJP (1-1) led to the highest mycelial growth, which was 16.2% greater than that of the control, while QS-MAP (1-1) achieved the highest yield. These results suggest that optimal pruning from *Morus alba, Ziziphus jujuba, Malus pumila*, and *Vitis vinifera* enhances mycelial growth and yield. This is consistent with prior studies (3, 26). Thus, orchard waste has a significant effect on the growth and yield of *L. edodes*.

MPP emerged as an outstanding substrate for the accumulation of crude protein in *L. edodes*. The MAP and QS-MAP (1-1) groups had the highest crude protein content (29.75–30.16%), while the QS-MPP (1-1) group exhibited the highest crude fiber content (1.19%). Conversely, the MPP and QS-MPP (1-1) groups had the lowest crude fat content (17.92 and 15.67%). ZJP had the highest crude polysaccharide content (1.37%). These results indicate that *L. edodes* is a high-protein, low-fat, low-fiber fungus with rich nutritional value, consistent with existing research findings (41).

The use of fruit tree pruning as a cultivation substrate also affected trace element accumulation. The addition of pruning improved the contents of potassium, iron, zinc, copper, sodium, manganese, and magnesium (Table 5). VVP was found to increase the levels of iron, zinc, and copper, which is consistent with the results observed in other studies (4, 5). Although the yield of mushrooms cultivated with 40% *Actinidia deliciosa* purning is relatively low, the mushrooms produced contain a higher concentration of alanine, reaching 1.18 g/100 g, which represented an increase of 12.7% compared to that of the control. Prior research indicates that alanine serves as a precursor for the synthesis of various substances, and alanine is also commonly used as a food additive to enhance sweetness, improve taste, and provide preservative effects. Additionally, alanine has applications in feed, pharmaceuticals, and the chemical industry (42, 43). Therefore, further optimization of the mushroom cultivation formula using kiwi fruit substrate is recommended to increase the alanine content. QS-VVP (1-1) as a substrate increased medicinal, umami, sweet, and bitter amino acids, while MAP increased several amino acid types but reduced SAAs. This aligns with previous research (4, 44). *Camellia oleifera* shells have also been used effectively as substrates for cultivating high-protein mushrooms (45).

Utilizing fruit tree pruning residues for mushroom cultivation not only addresses the issue of large quantities of pruning residues ending up as waste without suitable storage but also fully capitalizes on their value, converting waste into a resource, enhancing crop quality, and achieving both ecological and economic benefits (46). This study demonstrates that utilizing *Malus pumila, Vitis vinifera, Morus alba*, and *Ziziphus jujuba* pruning residues as alternatives to *Quercus* sawdust for cultivating *L. edodes* is viable. Our results thus provide a scientific foundation for the effective use of fruit tree pruning residues in mushroom cultivation.

## 5 Conclusion

An appropriate substrate formulation is a prerequisite for obtaining high yields of *L. edodes*. Different fruit tree branches have a significant impact on the growth of *L. edodes*. In areas where *Malus pumila, Vitis vinifera, Morus alba*, and *Ziziphus jujuba* trees are grown, it is recommended to use a substrate formed as a mixture of *Quercu*s sawdust and fruit tree pruning residues. The best formulation is 40% QS, 40% MPP, 17% bran, 1% sucrose, 1% CaCO_3_, and 1% gypsum. The second-best formulations are 40% QS, 40% VVP, 17% bran, 1% sucrose, 1% CaCO_3_, and 1% gypsum; 40% QS, 40% MAP, 17% bran, 1% sucrose, 1% CaCO_3_, and 1% gypsum; and 40% QS, 40% ZJP, 17% bran, 1% sucrose, 1% CaCO_3_, and 1% gypsum. VVP cannot be directly used for cultivating *L. edodes*, but can be utilized for extracting certain components from the mushroom.

Fruit tree pruning used as a raw material for cultivating *L. edodes* can enhance yield and quality to some extent. This study offers a reference for the substrate utilization of fruit tree pruning residues, possessing significant commercial value. Currently, we have implemented this practice in actual production in the corresponding fruit tree areas. The next step is to conduct large-scale trials in different regions and environments based on the existing research to verify the stability and effectiveness of the optimized cultivation formula.

## Data Availability

The original contributions presented in the study are included in the article/supplementary material, further inquiries can be directed to the corresponding authors.
